# Sustainable laser-based technology for insect pest control

**DOI:** 10.1038/s41598-021-90782-7

**Published:** 2021-05-26

**Authors:** R. Gaetani, V. Lacotte, V. Dufour, A. Clavel, G. Duport, K. Gaget, F. Calevro, P. Da Silva, A. Heddi, D. Vincent, B. Masenelli

**Affiliations:** 1 INSA Lyon, CNRS, Ecole Centrale de Lyon, Université Claude Bernard Lyon 1, CPE Lyon, INL, UMR5270, Univ Lyon, 69621 Villeurbanne, France; 2 INSA Lyon, INRAE, BF2I, UMR 203, Univ Lyon, 69621 Villeurbanne, France; 3 INSA Lyon, Ecole Centrale de Lyon, CNRS, Université Claude Bernard Lyon 1, AMPERE, UMR5005, Univ Lyon, 69621 Villeurbanne, France

**Keywords:** Zoology, Environmental sciences, Optics and photonics

## Abstract

Aphids damage directly or indirectly cultures by feeding and spreading diseases, leading to huge economical losses. So far, only the use of pesticides can mitigate their impact, causing severe health and environmental issues. Hence, innovative eco-friendly and low-cost solutions must be promoted apart from chemical control. Here, we have investigated the use of laser radiation as a reliable solution. We have analyzed the lethal dose required to kill 90% of a population for two major pest aphid species (*Acyrthosiphon pisum* and *Rhopalosiphum padi*). We showed that irradiating insects at an early stage (one-day old nymph) is crucial to lower the lethal dose without affecting plant growth and health. The laser is mostly lethal, but it can also cause insect stunting and a reduction of survivors’ fecundity. Nevertheless, we did not notice any significant visible effect on the offspring of the surviving irradiated generation. The estimated energy cost and the harmless effect of laser radiation on host plants show that this physics-based strategy can be a promising alternative to chemical pesticides.

## Introduction

Damage caused by insect pests on human cultures is a major plague. This impact of insects has become particularly critical in the last half century as the need to feed the rapidly growing world population has required the development of industrial agriculture and the monoculture model, which reduces crop diversity and favors pest proliferation^1^. Insect pest damage results in annual product losses to the agriculture industry estimated from 2 to 43% in Brazil, 25 to 43% in Asia and 20% to 90% in Africa depending on the nature of cultures^2–4^. Because of its efficiency and low cost, the use of chemical pesticides has become the main solution to control insect populations since the Second World War, particularly in Europe and the USA^5^. However, it is now established that these toxins, either through polluted groundwater or direct air intake, have alarming consequences to public health and the environment^6–11^, including a significantly increased risk of brain tumors^12^ and Parkinson’s disease^13^. In the “Ecophyto” plan, the French government announced the need to reduce pesticide use by half by 2018. Facing the failure to meet this commitment without ecologically sound alternatives, France has postponed the objective to 2025^14,15^.

Recently, several alternatives have been explored to reduce pesticide use but many of them rely either on non-specific (*i.e.* harmful to any insect without discrimination) or toxic chemistry-based strategies^16–18^. Bio-ecological methods have legitimately gained much interest and knowledge. They represent an important improvement strategy. However, some bio-ecological methods grant only a limited yield^19–21^. Besides these methods, complementary strategies stemming from other scientific areas than chemistry and biology are emerging. Among these, physics-based strategies are now being contemplated. A mechanical-based solution has been suggested using high-pressure water sprays^22^. This method can damage host plants and thus cannot be considered viable. Another uses electrical fields to repel or destroy pests but it is not specific to targeted insect pests and hardly applicable on large areas such as fields of agricultural crops^23^. Moreover, it requires high voltages (a few kV), which makes its use energy consuming and hazardous.

Few pioneering studies have been conducted looking into the control of small insects, such as cockroaches, mosquitoes, fruit flies and floor beetles, using laser systems from UV to IR^24–28^. A laser can deliver a collimated long-range high-power beam. It is therefore adapted to open-air operation conditions. UV light is well-known to induce DNA damage at high irradiances^24,25^. However, such a method is not suited for pesticide substitution since it requires a long time before the DNA damage can develop and lead to a global lethal effect over a large population (*e.g.* an irradiation of 48 h^24^, corresponding to a global delivered energy dose of 80 J cm^−2^). Moreover, provoking mutations to the DNA of insect population is not innocuous in the long term since it could lead to genetically modified and adapted species. In contrast, the use of pulsed (~ 25 ms) green laser light (532 nm wavelength) or CO_2_ IR (10.6 µm wavelength) lasers turned out to be effective to kill mosquitoes (*Anopheles stephensi*) with moderate energy doses (about 1 J cm^−2^)^25^.

Here, we have investigated the use of laser radiation for insect control as a part of a global program that includes an initial step to specifically detect insect pests by their spectral profiles. This strategy has been proposed and patented recently^29^, and is attracting interest on an European scale^30,31^. For this strategy to be ecologically sustainable, two criteria must be met. First, the chosen laser radiation must be efficiently absorbed by the pests, leading to a lethal effect for minimal supplied energy. Second, the energy must be delivered on a short-time scale (< 100 ms) so that a wide portion of a crop field can be treated in a reasonable time (one day).

**Figure 1:**
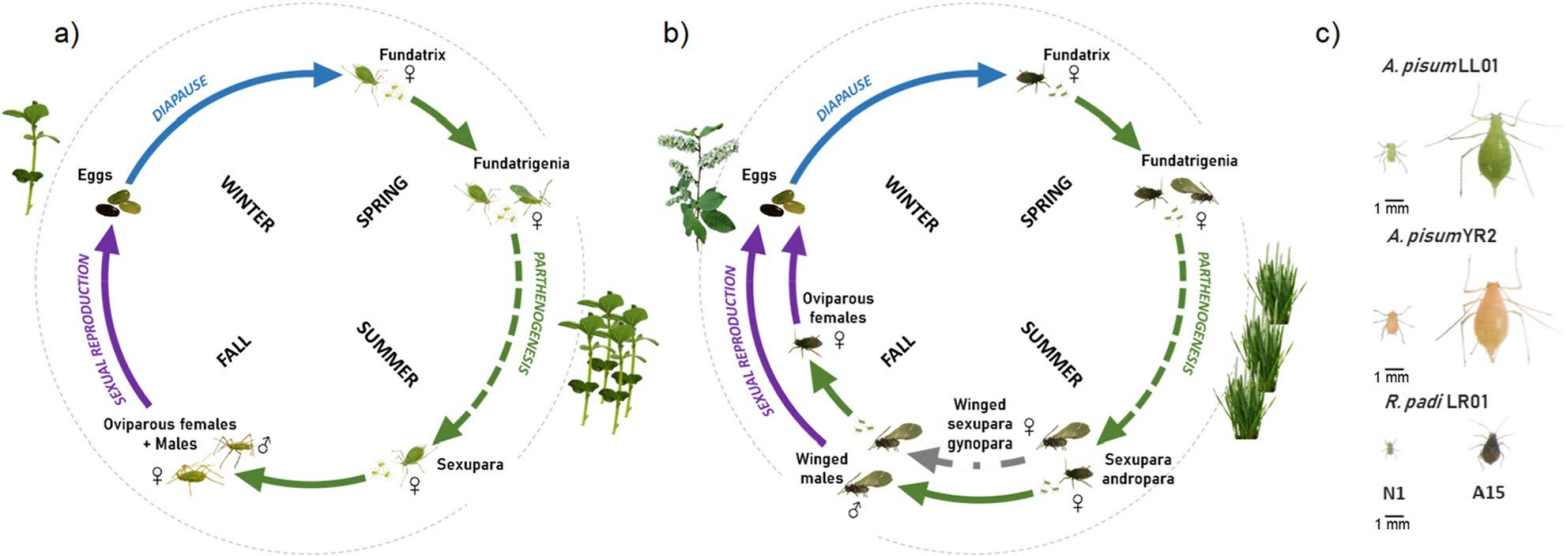
Aphids life cycles and morphology. (**a**) Monoecious holocyclic cycle of aphids *A. pisum* LL01 and YR2 lines^32^. (**b**) Dioecious holocyclic cycle of the aphid *R. padi*^33^*.* (b) Morphology of aphids *A. pisum* LL01 (green), YR2 (pink) and *R. padi* (black) at stages N1 (1d nymph) and A15 (15d adult). *R. padi* are 2 to 3 times smaller than *A. pisum* individuals. Details on aphid cycles are provided in the Supplementary Information section. Pictures taken by Virginie Lacotte (BF2i laboratory) except for sexual forms of the aphids and their eggs^34,35^ (from the Encyclop’Aphid website).

In the present study, we have focused on three distinct laser radiations; at 532 nm, 1070 nm and 10.6 µm. This is meant to probe the efficiency of two distinct mechanisms, namely absorption by the insect exoskeleton pigments and by the cell water, respectively. The first mechanism is species-dependent while the second is not. The goal was to determine the minimum energy required to kill 90% of a pest population in the minimum time. Aphids were chosen as insect pests as they are the most widespread and present a dangerous threat to cultures. Apart from being directly and indirectly extremely harmful to crops by feeding and spreading diseases, aphids are also a model system representative of other sap-sucking insect pests, including leafhoppers, mealybugs and whiteflies. We used three distinct lines belonging to two different aphid species: *Acyrthosiphon pisum* LL01 (green pea aphids), *Acyrthosiphon pisum* YR2 (pink pea aphids) and *Rhopalosiphum padi* (dark-green bird cherry-oat aphids). They offered the advantage of testing the laser on insects with different colors (and presumably with different exoskeleton properties) and of different sizes. Their life cycles are extremely complex and alternate, in the field, sexual and clonal parthenogenetic reproductions (Fig. 1), but all can be reared under obligate parthenogenesis in laboratory conditions. To be efficient and less energy consuming, any annihilation strategy thus requires treating the plants as early as possible and specifically in the early aphid development before they start reproducing clonally. Obviously, as mentioned before, a detection system that identifies the presence of pests in crop fields as soon as possible must be exploited before a selective lethal treatment is dispensed.

## Results and discussion

To assess a global strategy using a laser, the lethal dose to ensure the annihilation of 90% of the population (LD 90) after one day was first determined. To accurately retrieve this value, non-synchronized adult aphid (*A. pisum* LL01, *A. pisum* YR2 and *R. padi)* lines were used. The choice of the non-synchronized population is justified as being representative of what would be found in a field if no early detection system was coupled to the lethal system. Aphids were exposed to three distinct laser radiations, namely in the visible (532 nm wavelength), in the near infra-red (1070 nm) and in the far infra-red (10.6 µm). Based on the results obtained in this first experiment, we next focused on synchronized one-day-old nymph (N1) populations irradiated by a CO_2_ laser radiation of 10.6 µm wavelength. The corresponding LD 90 was measured at different times after irradiation. The effects on the irradiated aphids, as well as on their progeny, were investigated. This allowed us to establish the efficiency and energy effectiveness of the proposed strategy.

### Lethal dose (LD90) of non-synchronized adult aphids for visible and infrared wavelengths

**Figure 2:**
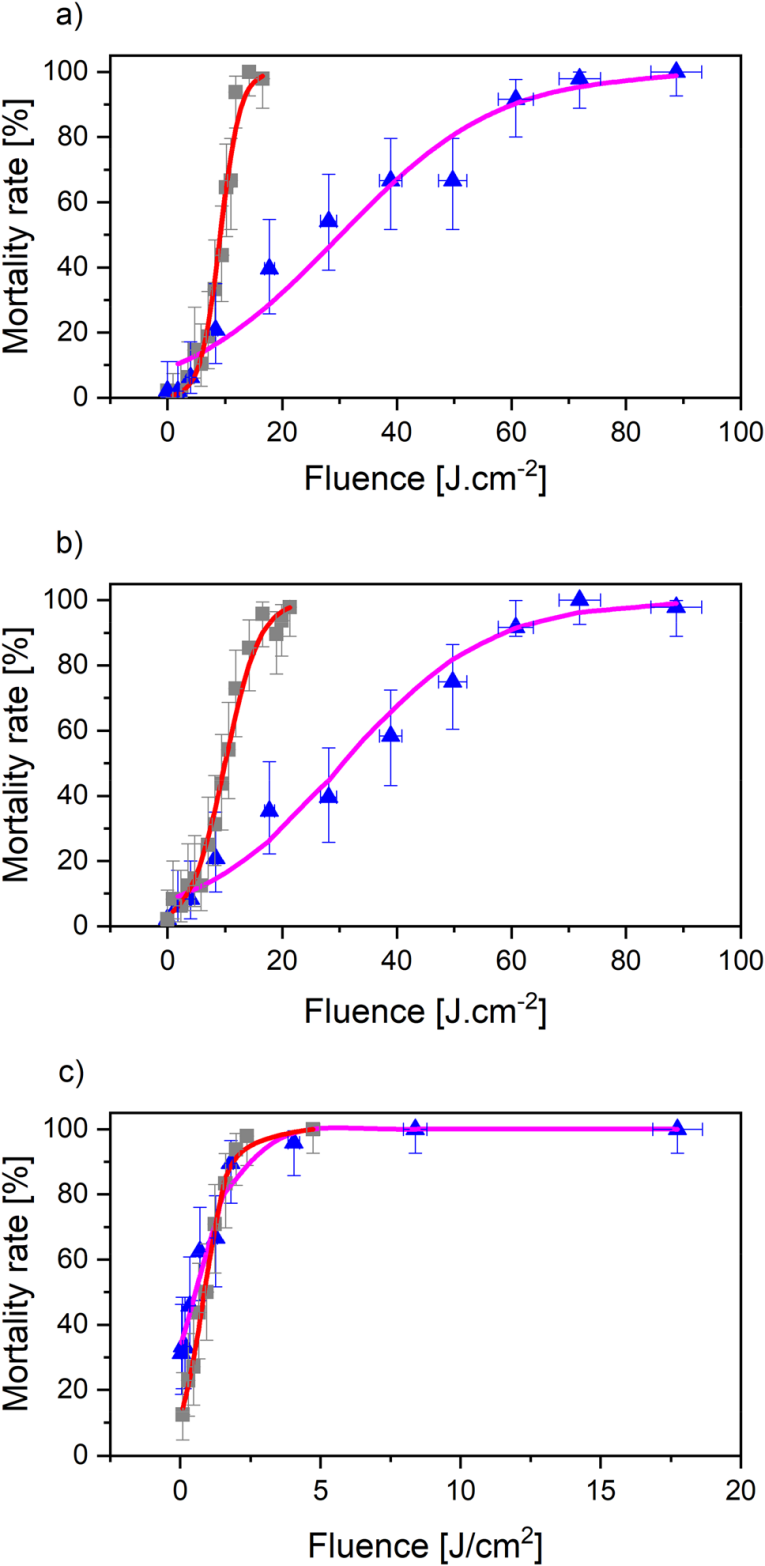
Mortality rates as a function of the fluence for 532 nm and 10.6 µm lasers at Day + 1. Error bars are plus and minus exact binomial 95% confidence intervals. Control samples showed that 96% or more aphids survived. (**a**) *A. pisum* LL01 (green aphids). (**b**) *A. pisum* YR2 (pink aphids). (**c**) *R. padi* (dark-green aphids). Blue dots and Gray squares refer to experiments done using the 532 nm and 10.6 µm lasers respectively. The purple and red solid lines are logistic regressions to the data obtained with the 532 nm and 10.6 µm lasers respectively. Statistical tests are available in Supplementary Table S1.

Mortality rates measured one day after irradiation as a function of the fluence at 532 nm and 10.6 µm for samples of 48 aphids (see “Methods” section) are displayed in Fig. 2. Curve fitting was achieved using a logistic regression. All of the statistical tests conducted showed that the logistic regression is the good model to fit the data (Supplementary Table S1). Lethal doses for 90% death rate in the populations (LD 90 values) extracted from logistic fits (Fig. 2) for the three aphid lines are gathered in Supplementary Table S2.

Both the green and CO_2_ lasers are efficient at killing the three aphid lines, with LD90 ranging from 1 to more than 50 J cm^−2^. However, the CO_2_ laser reduces the LD90 value by about 75% for *A. pisum* LL01 and *A. pisum* YR2, whereas it is nearly equivalent for *R. padi* with both lasers (reduction by only 21%). In addition, by selecting one wavelength, we noticed that *A. pisum* LL01 and *A. pisum* YR2 exhibit similar behavior, while *R. padi* remains the most sensitive to radiation, showing the smallest LD90. While *A. pisum* LL01 and YR2 both have the obligatory symbiont *Buchnera aphidicola* which participates in their nutrition, only YR2 has a secondary symbiont *Regiella insecticola* that can provide the host with more resistance to biotic and abiotic stresses (temperature, parasitoid attack, etc.)^36^. The similar results of *A. pisum* LL01 and YR2 aphids indicate that despite the presence of the secondary symbiont, aphids are equally sensitive to the new stress caused by the CO_2_ laser. Otherwise, the high sensitivity of *R. padi* outlines a better absorption in the visible and far-infrared range. Besides, the 1070 nm wavelength is inefficient for killing *R. padi* aphids, requiring lethal doses four orders of magnitude higher than the two other wavelengths (Supplementary Table S3 and Supplementary Discussion).

It was reported on other insects that laser induced damages are due to their optical absorption properties^25^. This implies that a better absorption coefficient leads to a higher temperature in the sample. The fundamental effect of lethality would thus be a rapid increase of the body temperature. Considering water contained in the cells to be the main cause of laser absorption, we can state that this is inefficient at 532 nm, somewhat efficient at 1070 nm, and very efficient at 10.6 µm. The absorption coefficient is a few 10^−3^ cm^−1^ at 532 nm, 10 cm^−1^ at 1070 nm and 1000 cm^−1^ at 10.6 µm^37^. Considering a 1 mm thick aphid (typical size of an *R. padi* specimen), much less than 1% of the 532 nm laser radiation entering the aphid is absorbed by the cells. Nonetheless, this fraction amounts to 60% and 100% at 1070 nm and 10.6 µm, respectively. Moreover, in the latter case, the radiation is absorbed over a typical depth of 50 µm. These estimations do not take into account the different reflectance for each wavelength due to the exoskeleton. Nevertheless, they partly explain why the 10.6 µm radiation is the most efficient.

If absorption by water is most likely the lethal cause at 10.6 µm, it is obviously not the case at 532 nm and 1070 nm. At both these wavelengths, absorption by aphid pigments is a more probable mechanism. Besides, if we consider the reflectance of YR2 and LL01 *A. pisum* aphids as obtained from hyperspectral measurements (Supplementary Fig. S1), we note that reflectance at 532 nm is about 0.35 or 0.4 for aphids, depending on their color. For wavelengths over 1000 nm, the reflectance is twice as large (about 0.75). This means that only half as much of the incident energy is available for pigment absorption at 1070 nm as compared to 532 nm. This is in qualitative agreement with the observed inefficiency of the 1070 nm laser to destroy aphids.

So far, we did not consider the time of energy delivery as a parameter. However, using ultra-short pulses with identical energy, very large peak powers may result in significant changes, as has already been proven at smaller length scales (scale of bacteria) ^38^. When dealing with pigment absorption, it might be beneficial. On the contrary, when the absorption occurs in water, as the heat penetration formula is proportional to the square root of the time $t$, reducing $t$ by a factor of two results in a decrease in penetration depth of more than 30%. It will consequently be less harmful.

### Mortality rate evolution and transgenerational effects in synchronized green aphids

#### N1 mortality rate evolution

LD90 values of about 15 J cm^−2^ previously found for non-synchronized populations of *A. pisum* might seem already satisfying. However, in order to make the pest control system faster, more energy efficient and secure, any reduction of the minimum lethal dose has to be sought. This can be expected if one considers a synchronized population. In addition, if we relax the constraint of killing 90% of a population after only one day and impose the same mortality rate after a longer period, the minimum lethal dose could be lowered even further.

N1 nymphs were selected because they are the youngest and thus presumably the weakest population among the synchronized ones. The *A. pisum* LL01 nymphs (green aphids) were chosen as representative aphids because they are as sensitive as *A. pisum* YR2, and less sensitive than *R. padi*. Furthermore, the life history traits of the parthenogenetic morphs of this aphid line in the laboratory conditions used in this work are well documented in the literature^39^. This will help to avoid any under-estimation of the LD90 for N1 populations. We considered the LD90 dose seven days after irradiation (Day + 7-timescale), and compared it to the LD90 dose one day after irradiation (Day + 1-timescale). This is justified by the development time of aphid nymphs.

**Figure 3:**
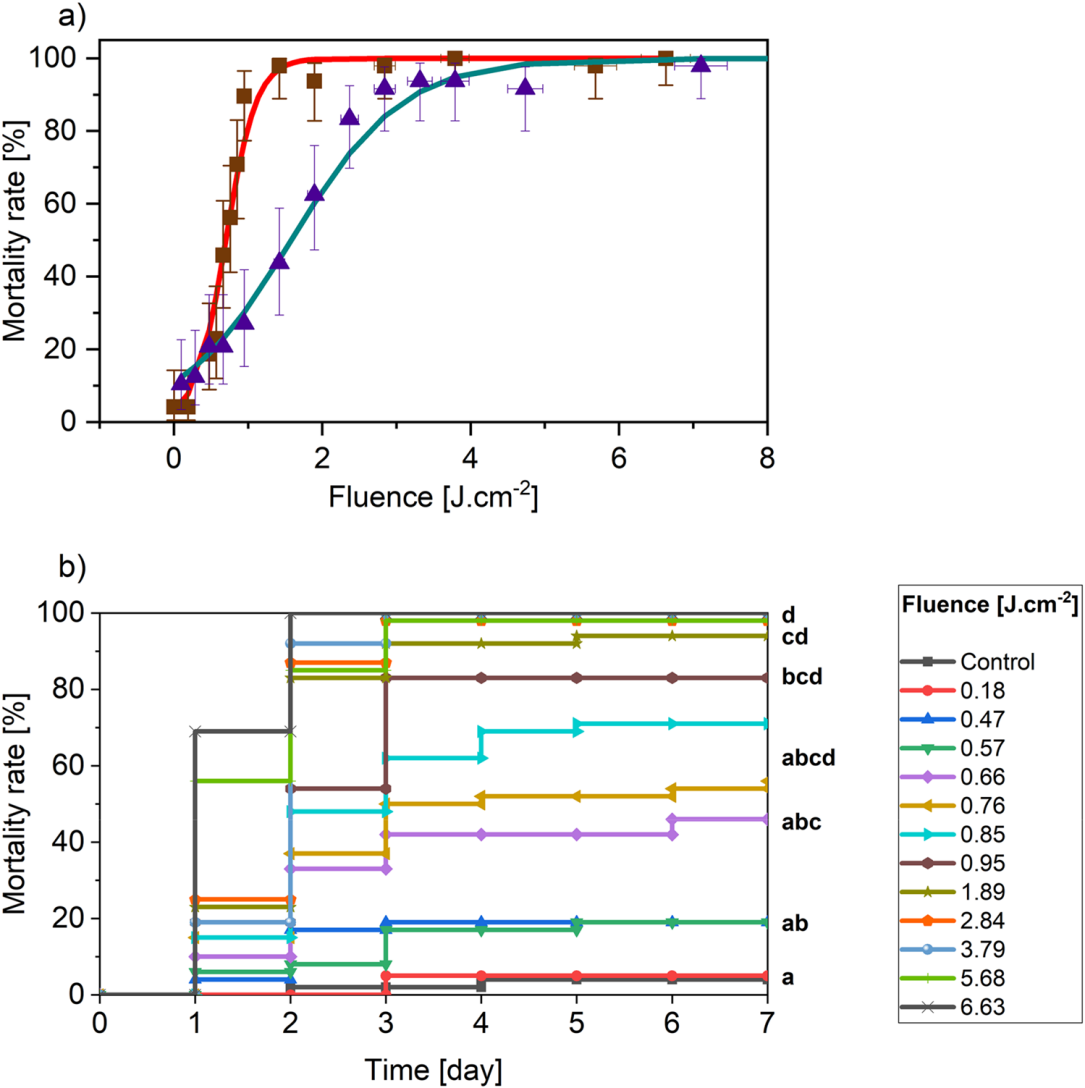
(**a**) Mortality rates as a function of the fluence using a 10.6 µm laser at Day + 1 (Blue triangles) and Day + 7 (Red squares). Control samples showed that 96% or more aphids survived. Error bars are plus and minus exact binomial 95% confidence intervals. The gray and red solid lines are logistic regressions obtained at Day + 1 and Day + 7 respectively. Statistical tests are available in Supplementary Table S4. (**b**) Evolution of the mortality rate over seven days at different fluences using the CO_2_ laser on synchronized N1 *A. pisum* LL01 nymphs (green aphids). Letters in the right refer to the results of the 95% confidence interval GLM Gaussian test.

Figure 3a displays the mortality rate as a function of the fluence using the CO_2_ laser at Day + 1 and Day + 7. The retrieved LD90 value at Day + 1 is four times lower (3.29 J cm^−2^), compared to the value found for adults of the same species (12.91 J cm-2). Moreover, this same value is lowered by a supplemental factor of 2.6 when going from a Day + 1 to a Day + 7-timescale, reaching the minimum value of 1.15 J.cm^−2^. Finally, going from adults at Day + 1 to N1 at Day + 7 lowers the LD90 value by more than ten times. Nymphs appear to be more sensitive than adults, so treating the aphids at this development stage and at Day + 7 is of great interest if one wants to build an energy-efficient pest control system based on this method.

Moreover, a closer analysis of the mortality rate evolution (Fig. 3b) revealed that irradiation effects are not linear. First, we can roughly distinguish three categories of samples. Those irradiated with a fluence less than or equal to 0.57 J cm^−2^ are hardly affected and their mortality rate remains insignificant. For the samples irradiated with a fluence between 0.57 and 0.95 J cm^−2^, the laser radiation produces significant defects but is not enough to achieve a mortality rate of 90%. Over a fluence of 0.95 J cm^−2^, the 90% mortality rate is achieved. Second, for the latter samples, a clear threshold is observed at Day + 3 timescale. Three days after the irradiation, the change of the mortality rate is minor. This shows that, on the one hand, the minimal energy necessary to destroy N1 *A. pisum* LL01 is 1.15 J cm^−2^ and, on the other hand, the shortest time after which one can perform a second treatment is after a delay of three days.

#### F0 and F1 generation effects

**Figure 4:**
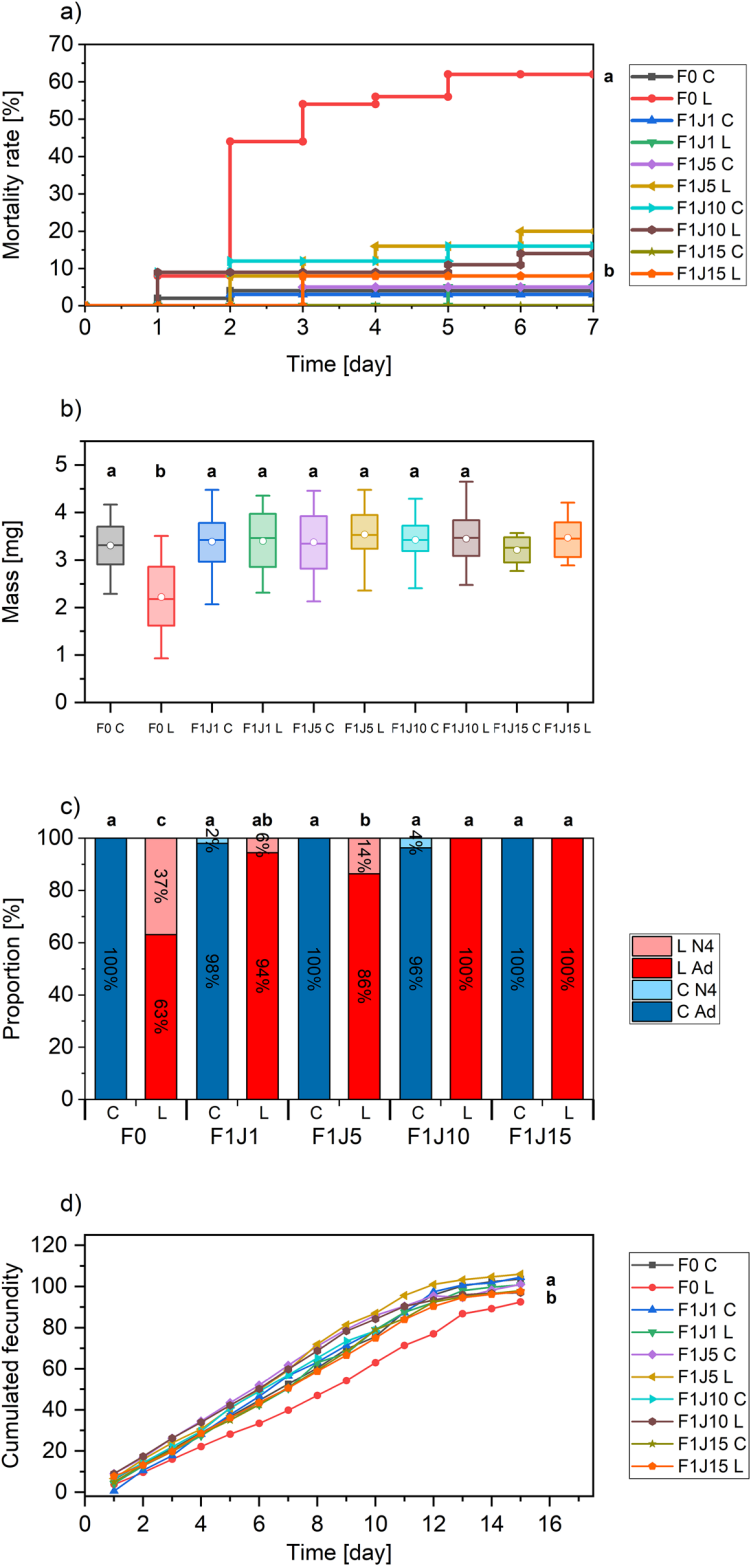
(**a**) Mortality rates over time for the first (F0) and second (F1) aphid generations. F0 C refers to the untreated first generation, while F0 L refers to the irradiated first generation. F1 JX refers to the second generation born the 1st, 5th, 10th and 15th day during the F0 reproduction period (95% interval confidence GLM Gaussian test). (**b**) Aphids’ mass (mg) at day seven (95% interval confidence ANOVA test; sample size F1J15 too small for statistics). (**c**) Proportion of adults (Ad) aphids in samples at day seven. N4 refers to the fourth stage of nymph, which precedes the adult stage (95% interval confidence χ^2^ and Fisher tests). (**d**) Evolution of cumulated fecundity (95% interval confidence GLM Poisson test).

Once the optimal conditions for total pest destruction have been characterized, we have wondered whether a non-lethal dose can affect the physiology of aphids and influence their fecundity and intrinsic rate of population increase, as several biotic and abiotic stresses can influence these parameters^40^. Even in the case where irradiated aphids can reproduce and give birth to nymphs of second generation (F1), the laser irradiation could also affect this F1 generation. To this end, we have examined biological parameters indicative of the health status of aphids (detailed in the “Methods” section) on F0 and F1 aphid generations (born the 1st, 5th, 10th and 15th day during the F0 reproduction stage) and on their offspring (Fig. 4).

A LD50 fluence of 0.76 J.cm^−2^ corresponding to approximately 50% of mortality (Fig. 3b) was selected. Our assumption was that 50% of N1 population surviving the chosen dose could be affected in such a way that their development could be impaired and their offspring reduced. We observed that, when irradiated at that fluence, N1 individuals could still generate nymphs via parthenogenesis. Only the F0 nymphs that received the treatment showed significant mortality while their F1 offspring exhibited no mortality (Fig. 4a). At day seven of development (end of the nymphal development), a significant weight reduction was observed for the irradiated sample (F0 L). However, their offspring F1 showed no difference with the control, attesting that fluence of 0.76 J cm^−2^ does not influence offspring’s weight.

The reduction of weight on the irradiated F0 aphids comes with a developmental delay (Fig. 4c). While all the untreated individuals of the first generation (F0 C as Control) reach the adult stage, only 63% do so in the irradiated first-generation population (F0 L as Laser), the rest remaining at the end of the nymphal period (N4). The development of the F1 L nymphs is no different from the control, except those laid on D1 and D5 of which 6 and 14% were not yet adults. It is possible that the first offspring of irradiated F0 aphids have a slight delay in development.

Finally, even if the irradiated F0 aphids can still reproduce, their fecundity is also impacted (Fig. 4d and Supplementary Fig. S2). Again, only the irradiated (F0 L) population is affected with a slight decrease of fecundity and intrinsic rate of increase (r_m_). This reduced fecundity is independent of the stunting observed previously since all the F0 aphids chosen to lay the F1 generation were adults at D0. However, the F0 L adults were smaller than the control adults (30% reduced weight), which could influence their production of embryos. The cumulated fecundity for the irradiated population (F0 L) after fifteen days is 11% lower than the control (F0 C) and its intrinsic rate of increase is 0.327 compared with 0.348 for the control.

In view of these results, we can theorize that laser treatment only influences irradiated aphid populations and not the next generations. Indeed, their offspring only shows a slight delay in reaching their maturity and their survival is not compromised.

#### Global strategy against pests

In order to be a reliable solution, a laser-based method must meet several criteria. First, the laser radiation must be efficient to kill the targeted insect. We assess this in “Introdcution” and “Results and discussion” sections. The use of CO_2_ laser radiation proves to be effective. The underlying mechanism, based on heating water contained in the cells, is a generic one. Indeed, prior research has found a LD90 value for mosquitoes of about 1 J cm^−2^ therefore quite close to ours using the same wavelength^25^. Targeting the water of the cells is more robust than targeting pigments of insects.

Second, the laser-based destruction of pests must be energy efficient. In order to estimate the actual energy delivered by the laser while shooting, we have considered the average area for a French sugar beet field of 17.6 ha with a row every 50 cm and a plant every 25 cm representing 13.6 million of plants per field^41^. Furthermore, we suppose that the early detection of N1 aphids allows one to treat only 5% of plants with one hundred aphids killed per plant. Based on the aforementioned LD90 values, this gives a total delivered energy of 200Wh per field. Obviously, this is a minor part of the energy cost estimation. Actually, the laser consumes 200 W while shooting and 150 W in stand-by. If, as assumed, the early detection allows to treat a small fraction of the field, the laser will be in stand-by mode most of the time. Consequently, the energy used will ultimately depend on the velocity of the chosen mobility vector. This point is out of the scope of the present study. However, it has been addressed first in a patent^29^ held by the GreenShield Technology start up and more recently investigated by the consortium of academic and industrial laboratories in the framework of the EU project WeLASER^31^. Both studies highlight that a strategy combining a sensitive early detection method and a subsequent laser-based destruction technique can be economically and energetically sustainable.

Third, the strategy has to be ecologically sustainable. It must be robust with respect to a false positive event. We must thus ensure that laser radiation hitting the plant host and not an aphid does not damage the plant so as to threaten its life or growth. For that purpose, we have shot several plant organs of *Vicia faba* and *Triticum aestivum* that are our aphid host plants, using LD90 values for both aphid species at different stages (N1 or Adult) and days (D + 1 or D + 7). We used samples of 20 plants per targeted plant organ and per fluence. The results show that the fluences we use are harmless for their growth (Methods, Supplementary Discussion, and Supplementary Fig. S3, S4 and S5).

However, since the CO_2_ laser acts on the water contained in the cells, the fluences used are probably harmful to non-target insect community (like aphid predators for instance). To avoid this collateral damage effect, the hyperspectral identification of insects present in the fields must be as accurate as possible, prior to the laser treatment.

## Conclusion

We have analyzed the LD90 for three aphid species using a visible and an IR laser. The CO_2_ IR laser, giving rise to the lowest LD90, proves to be the most suited. Its mechanism of interaction with living matter makes it also adapted to all pests. Our study also shows that the minimum LD90 is obtained first by irradiating nymphs instead of adults and second by considering a lethal date seven days after the irradiation instead of one day. However, the laser is only effective on directly irradiated aphids and not on the offspring issued from the irradiated generation. Hence, the laser is an effective solution against aphid proliferation, particularly when coupled with an early treatment of aphids. We also have estimated the energy cost and showed that the laser effect on host plants in case of a false positive detection is negligible. Thus, a global strategy based on a laser destruction of pests can be considered sound.

## Methods

### Insects and plants

*Acyrthosiphon pisum* individuals are from parthenogenetic females from two field-originated lines, LL01 and YR2 (Supplementary Fig. S6). The two lines were maintained on young broad bean plants (*Vicia faba*, L. cv. Aquadulce), in a phytotron at 21 °C ± 1 °C and 65% RH ± 5% with a 16 h photoperiod. Sampling of synchronized N1 aphids was performed as described in^39^.

*Rhopalosiphum padi* individuals LR01 (Supplementary Fig. S7) have been reared on wheat seedling (*Triticum aestivum* [Linnaeus] cv. Orvantis) in the same controlled conditions than *A. pisum*.

In order to get close to field conditions, non-synchronized parthenogenetic adults were first used for the radiation experiments.

Organic seeds of *Vicia faba*, L. cv. Aquadulce and *Triticum aestivum* [Linnaeus] cv. Orvantis were supplied by Graines-Voltz® (Loire-Authion, France) and Nature et Progrès® (Alès, France), respectively. The seeds were germinated in commercial peat substrate Florentaise® TRH400 (Saint-Mars-Du-Désert, France) in a phytotron using the same controlled conditions as for aphid rearing. The collection of plant material in this manuscript complies with relevant institutional, national and international guidelines and can be obtained from various seed companies worldwide.

### Optical set up

The experiment used three different lasers. A 2.4-mm-beam-diameter CO_2_ pulsed laser manufactured by Access Laser, a 1-mm-beam-diameter DPSS CW 532 nm laser manufactured by CNI Lasers and an 80-µm-beam-diameter pulsed laser at 1070 nm manufactured by IPG. A red pointer was associated with them in order to aim at the targets more easily, especially for CO_2_ laser pulses. Laser beams were brought to aphids using a two mirrors-movable system operated by a Jetson GPU from NVIDIA linked to an Arduino Uno card to control laser triggering (Supplementary Fig. S7). The command system triggered a time-regulated laser impulsion through the optical system that reached the aphids’ abdomen. Upstream, we monitored command signals on an oscilloscope to check its shape and frequency.

Apart from the nature of the aphids (*A. pisum* LL01*, A. pisum* YR2 and *R. padi*), the variables were laser fluence controlled by changing the impulsion time, the Pulse Width Modulation (PWM) for the CW laser (through external command), the number of shots for pulsed lasers. The 532 nm laser cannot reach impulsion time shorter than 5 ms due to its technical characteristics making the beam inconsistent under this value. Changing the PWM allowed exploring the fluence under this impulsion time. An assumption made here is that changing the PWM is equivalent to reducing the laser impulsion time and that only the average power matters. We choose impulsion time and frequency for the CO_2_ lasers as 400 µs and 750 Hz, respectively, in order to have the maximum average power. These values were not changed during experiments each time using this laser. Thus, the overall energy deposited by this laser was monitored through the number of delivered pulses.

Laser beam power is measured a posteriori thanks to S425-C sensor and the PM100D console manufactured by ThorLabs with a more or less 5% error announced by the manufacturer^42^.

A FX10 hyperspectral camera from Specim® have been used to measure the aphids light reflectance spectrum between 400 and 1 000 nm. Hyperspectral measurements were made on 15 days-old adults of the three aphid lines and repeated three times.

### Mortality counts

Aphids were collected from plants, treated with the laser and then put into a translucent box with leaves changed after one day to avoid death by food insufficiency or directly on *Vicia faba* plant depending of the duration of the monitoring. We observed each aphid after one day or every day for longer period, depending on the experiment timescale (see Supplementary Fig. S9). Examinations allowed aphids discrimination in three categories: alive, sick and dead. Examination was tricky because aphids often retracted their feet and immobilized themselves when stressed. In addition, under the heat generated by the laser beam, the abdomen could darken, probably because of melanization. It does not necessarily mean that the aphid is dead. Hence, examinations must be done very meticulously, especially when performed with the smallest aphids (N1): when N1 aphids looked sick, their status were reexamined one day latter to ensure whether the insect was dead or still alive and able to recover. Pushing forward aphids by pressing on their abdomen to make them move or stack two or three aphids were useful maneuvers because they resulted in a prompt or quasi-prompt reaction from alive or sick aphids. As long as an aphid moved, even only slightly its feet, it was considered alive or sick depending on its behavior. Alive aphids can move quite quickly and can overturn when set on their back. On the contrary, sick aphids barely move and hardly or cannot overturn properly when set on their back. Furthermore, dead aphids are those with a much-darkened abdomen, spread stiff feet and do not react to any stimulus.

### N1 *A. pisum* LL01 population study protocol

This protocol aimed at retrieving the energy inhibiting the most aphid development using the minimum energy. To this end, we first have irradiated samples of 48 N1 *A. pisum* LL01 nymphs with the CO_2_ laser. Fluences applied came from those applied on *R. padi* adults because of their size similitude. For each sample, we sowed *V. faba* eleven days before depositing aphids. Therefore, these were big enough to receive the sample. *A. pisum* specimens were deposited on plants after laser irradiation. We reviewed mortality daily for seven days, using the same method described in “Mortality counts” section. This seven-day supervision resulted in the time evolution of the mortality rate (Fig. 3b).

Secondly, we have investigated laser-induced effects on first- and second-generation aphids during a biological monitoring (Supplementary Fig. S8 and S9). For each modality (F0 C, F0 L …), the samples were made of 50 synchronized N1 *A. pisum* LL01 nymphs splitted into 5 repetitions of 10 aphids per cage containing a plant. We chose to apply a 50%-lethality fluence (LD50). Deposited N1 nymphs achieved their development cycle (seven days) and reproduction cycle (fifteen days) during the biological monitoring (Fig. 1). Mortality was assessed every day. After seven days or more, aphids reached their adult stage depending on laser damages. We, therefore, measured their mass and count developmentally delayed aphids. *V. faba* were changed once they had reached their maximum development stage.

Then, one adult aphid per repetition corresponding to five aphids among the samples of fifty aphids were randomly taken and deposited on a separated plant. Thus, we measured every day during fifteen days their fecundity by counting the number of offspring and their health state (mortality and deformity). The nymphs laid at day one (F1J1), five (F1J5), ten (F1J10) and fifteen (F1J15) were again deposited on a new plant. As before, we monitored their development during seven days followed by the fecundity of five randomly chosen aphids during fifteen days. We also calculated the intrinsic rate of increase of aphids for every treatment using the equation r_m_ = 0.74 (ln M_d_)/T, with T as the time from birth to the start of reproduction, M_d_ as the reproductive output per aphid during a period equal to T, and 0.74 as a correction factor^40^ (Supplementary Fig. S2). Hence, the biological monitoring was performed on two generations (Fig. 4).

### Laser induced effects on infested plants protocol

This protocol aimed at reproducing a shot irradiating directly the plant due to a false positive detection or a shot imprecision. We monitored *Vicia faba* and *Triticum aestivum* whose are host plants for *A. pisum* and *R. padi* respectively during seven days. Each plant was kept in the phytotron with the same experimental conditions applied before (21 °C ± 1 °C, 65% RH ± 5%).

Thus, each sample contained 20 seedlings per fluence and per organ targeted. According to aphids on their host plants, we irradiated the adaxial (up) and abaxial (under) surfaces of the leaf, the apex and the stem. We targeted four times the same organ. To avoid the underestimation of plant damage, we have applied fluences corresponding to LD90 values for adults or N1 at 1 or 7 days (Supplementary Table S5) with the CO_2_ laser.

The plants were pictured every day and biological parameters, including height, weight and leaf surface, were measured on the seventh day (Supplementary Table S6).

### Statistical tests

Logistic fits have been already used in similar studies^25^. Statistic tests were done on every fit to ensure that the logistic model corresponded to the experimental results. The first one is the log-likelihood ratio and the pseudo-R^2^. For instance, for *A. pisum* YR2 aphids irradiated with the CO_2_ laser, the obtained values are 0.40 and 0.57. Moreover, the Hosmer–Lemeshow test (HLT), the area under the curve (AUC) of the Receiver Operating Characteristic (ROC) curve are other tests performed. The HLT is a statistical test for goodness of logistic fit. ROC curve shows the true positive rate over the false positive rate of the model and the closer this curve is to one, the better the fit. Using the same previous example, the HLT is passed and the AUC value is 0.91, which shows that the fit is accurate and correct.

Statistical test results for the other wavelengths and species are available (Supplementary Table S1, 4). For the 532 nm laser experiment done with adults *A. pisum* LL01, we add “Before readjustment” and “After readjustment” labels because the Hosmer–Lemeshow test was failed and the sample at 50 J cm^−2^ seemed to be abnormal. This sample was removed to check whether it distorted the test. The fit was barely changed but when the HLT was done, it was used to prove that the fit was good but disturbed by this experimental sample. This is something to watch out when using the HLT because a single aberrant experimental point can make the test failed.

During the N1 green aphids (*A. pisum* LL01) population study, all the treatments were repeated five times. Statistical tests were performed using R Studio software. All statistical tests were carried out with a significance level of 5% (0.05). The daily measurements, which showed a linear change, were tested using the GLM linear regression model with Gaussian or Poisson regression after observation of the residuals (daily mortality and fecundity). The continuous quantitative measurements taken at day 7 on large samples (aphid mass, plant mass, plant height) have been evaluated with a Shapiro–Wilk normality test followed by a Levene homogeneity of variance test and an ANOVA comparison of means followed by a test of Tukey HSD. Finally, measurements relating to a proportion such as the adult aphid rate were analysed with the χ^2^ ratio comparison test 2 to 2 or with Fisher's exact test for smaller samples.

## Supplementary Information

Supplementary Information.
