# Supplementary material for: Sustainable laser-based technology for insect pest control
Source: Sci Rep. 2021 May 26;11:11068. doi: 10.1038/s41598-021-90782-7 (PMC8155209; doi:10.1038/s41598-021-90782-7)
Supplement: Supplementary file 1 — Supplementary Information. [file 41598_2021_90782_MOESM1_ESM.docx]

**Sustainable laser-based technology for insect pest control**

R. Gaetani^1^, V. Lacotte^2^, V. Dufour^3^, A. Clavel^2^, G. Duport^2^, K. Gaget^2^, F. Calevro^2^, P. Da Silva^2^, A. Heddi^2^, D. Vincent^1^, B. Masenelli^1*^

^1^ Univ Lyon, INSA Lyon, ECL, CNRS, UCBL, CPE Lyon, INL, UMR5270, 69621 Villeurbanne, France

^2^ Univ Lyon, INSA Lyon, INRAE, BF2I, UMR 203, 69621 Villeurbanne, France

^3^ Univ Lyon, INSA Lyon, ECL, CNRS, UCBL, AMPERE, UMR5005, 69621 Villeurbanne, France

* Corresponding author: bruno.masenelli@insa-lyon.fr

**Supplementary Tables**

a)

|  | **532 nm** | | **10.6 µm** |
| --- | --- | --- | --- |
|  | **Before readjustment** | **After readjustment** |  |
| **Log-Likelihood Ratio** | 0.41 | 0.46 | 0.47 |
| **Pseudo-R^2^** | 0.58 | 0.63 | 0.63 |
| **AUC** | 0.92 | 0.94 | 0.93 |
| **Hosmer-Lemeshow** | 🗶 | ✓ | ✓ |

b)

|  | **532 nm** | **10.6 µm** |
| --- | --- | --- |
| **Log-Likelihood Ratio** | 0.43 | 0.40 |
| **Pseudo-R^2^** | 0.60 | 0.57 |
| **AUC** | 0.93 | 0.91 |
| **Hosmer-Lemeshow** | ✓ | ✓ |

c)

|  | **532 nm** | **10.6 µm** |
| --- | --- | --- |
| **Log-Likelihood Ratio** | 0.31 | 0.36 |
| **Pseudo-R²** | 0.45 | 0.52 |
| **AUC** | 0.87 | 0.90 |
| **Hosmer-Lemeshow** | ✓ | ✓ |

**Supplementary Table 1: Irradiation tests on adult aphids: summary of the statistical. a) *A. pisum* LL01. b) *A. pisum* YR2. c) *R. padi*. For the 532 nm laser experiment done with the *A. pisum* LL01, we add “Before readjustment” and “After readjustment” labels because the Hosmer-Lemeshow test failed and the sample at 50** $\boldsymbol{J/}\boldsymbol{cm}^{\boldsymbol{2}}$ **seemed to be abnormal.**

| **Wavelength (nm)** | **LD90 for *A. pisum* LL01** **at Day+1 (J.cm^-2^)** | **LD90 for *A. pisum YR2* at Day+1 (J.cm^-2^)** | **LD90 for *R. padi* at Day+1 (J.cm^-2^)** |
| --- | --- | --- | --- |
| **532** | 53.55 | 58.54 | 2.29 |
| **1070** | N.A | N.A | N.A |
| **10600** | 12.91 | 16.56 | 1.80 |

**Supplementary Table 2: Summary of LD90 values at Day+1 for three wavelengths for *A. pisum* LL01*, A. pisum* YR2 and *R. padi* adults. Samples of 48 aphids were used for statistical robustness.**

| **Aphid Species** | **Pulse energy (mJ)** | **Number of shots** | **Corresponding fluence (kJ.cm^-2^)** | **Commentary** |
| --- | --- | --- | --- | --- |
| *R.padi* | 0.875 | 10000 | 170 | 12/12 aphids killed |
| *R.padi* | 0.875 | 5000 | 85 | 3/12 aphids killed |
| *R.padi* | 0.875 | 3000 | 51 | Winged female killed, Female alive |
| *R.padi* | 0.875 | 1000 | 17 | 0/12 aphids  killed |

**Supplementary Table 3: Summary of data for the 1070 nm laser experiment on *R. padi*. Samples of 12 aphids were used.**

|  | **Day+1** | **Day+7** |
| --- | --- | --- |
| **Log-Likelihood Ratio** | 0.42 | 0.50 |
| **Pseudo-R²** | 0.59 | 0.64 |
| **AUC** | 0.92 | 0.94 |
| **Hosmer-Lemeshow** | ✓ | ✓ |

**Supplementary Table 4: Summary of statistical tests on N1 at Day+1 and Day+7**

| **Plant** | **Number of plants** | **Fluence [J.cm^-2^]** | **Number of impact on each organ** | **Targeted organ** |
| --- | --- | --- | --- | --- |
| *Vicia faba* | 80 | **16.56** (LD90 Day+1 Adult *A. pisum* YR2) | 4 | -Apex  -Stem  -Adaxial leaf surface  -Abaxial leaf surface |
| *Vicia faba* | 80 | **1.15** (LD90 Day+7 N1 *A. pisum LL01*) | 4 | -Apex  -Stem  -Adaxial leaf surface  -Abaxial leaf surface |
| *Triticum aestivum* | 60 | **1.80** (LD90 Day+1 Adult *R. padi*) | 4 | -Stem  -Adaxial leaf surface  -Abaxial leaf surface |

**Supplementary Table 5: Experimental conditions for laser induced effects on host plants protocol.**

| **Observation/Measurement** | **Detail** | **Frequency** |
| --- | --- | --- |
| Impact healing | Photography of impacts | Every day |
| Height | Aerial part from collar to apex [cm] | On the seventh day |
| Wet mass | Aerial wet mass [g] |  |
| Dry mass | Aerial dry mass [g] |  |
| Leaf surface | Total leaf surface [cm²] |  |

**Supplementary Table 6: Observations and measurements for the laser induced effects on host plants protocol.**

**Supplementary Information about Figure 1 legend**

**Aphids’ life cycles**

For *A. pisum* aphids, after mating during the fall, oviparous sexual females deposit overwintering eggs. During the spring months, the fundatrix female hatched from the egg gives birth to live clonal fundatrigenia by parthenogenesis. These offspring undergo four molts during nymphal development to become unwinged or winged asexually reproducing adults. Winged individuals, capable of dispersing to new plants, are induced by crowding or stress during prenatal stages. After numerous cycles of parthenogenetic reproduction in summer, shorter autumn day lengths trigger the birth of sexual female and male aphids, which restart the cycle (Ogawa *et al.*, 2014, DOI [10.3389/fphys.2014.00001](https://dx.doi.org/10.3389%2Ffphys.2014.00001)). *A. pisum* lives exclusively on *Fabaceae*. The life cycle of the dark-green bird cherry-oat aphids is even more complex as this aphid uses the red birch *Prunus padus* as primary host for sexual reproduction and *Poeaceae* as secondary hosts during parthenogenesis. (Peng *et al*., 2017, doi: [10.1002/ece3.2720](https://dx.doi.org/10.1002%2Fece3.2720)). Both *A. pisum* and *R. padi* can be kept under obligate parthenogenesis in laboratory conditions.

**Supplementary Discussion**

***R. padi* results after 1070 nm irradiation**

The 1070 nm wavelength is inefficient for killing *R. padi* aphids as compared to the two other wavelengths for which a much weaker fluence is lethal. Since it takes fluences of almost 100 kJ.cm^-2^ at 1070 nm, the order of magnitude for lethality seems to be 10^4^ larger in the present case. We did not obtain enough data to extract solid statistical results so the logistic regression was not applied in this specific case. Moreover, only *R. padi* were tested because, based on the observations performed using green and IR lasers, they are more sensitive to radiations.

Peculiar to that wavelength, winged females and wingless females did not behave uniformly, winged females being more sensitive to the radiation than the second ones. We have no clear explanation for this phenomenon, but we noticed that this difference arise in the head area where pigments are the most abundant. This difference does not seem to come from body thickness.

**Laser induced effects on infested plants results**

Lesions are visible a few hours after irradiation and still present after seven days whatever the fluence chosen (Supplementary Fig. 2). However, no lesion can be observed on stems thanks to its thickness at 1.80 J.cm^-2^ and 1.15 J.cm^-2^ (LD90 Day+1 Adult *R. padi* and LD90 Day+7 N1 *A. pisum* respectively).

Moreover, after seven days, plants have started to heal which is visible by the darken leaves area, the drying out of dead cells and the deformation of irradiated organs. Irradiation on the apex causes the deformation of leaves visible as the plant develops.

*V. faba* plants treated with the LD90 Day+7 N1 *A. pisum* fluence have a slight lesion nearly non visible which heal at the surface. The apex displays also a minimum deformation and only visible on one or two leaves. Hence, the lower the fluence, the less intense the symptoms.

Although, these lesions caused by the laser do not influence growth of plants (Supplementary Fig. 3 and 4). Compared to control samples, there is no clear difference when height, mass, foliar area are compared in spite of a heterogeneity in samples.

**Supplementary Figures**


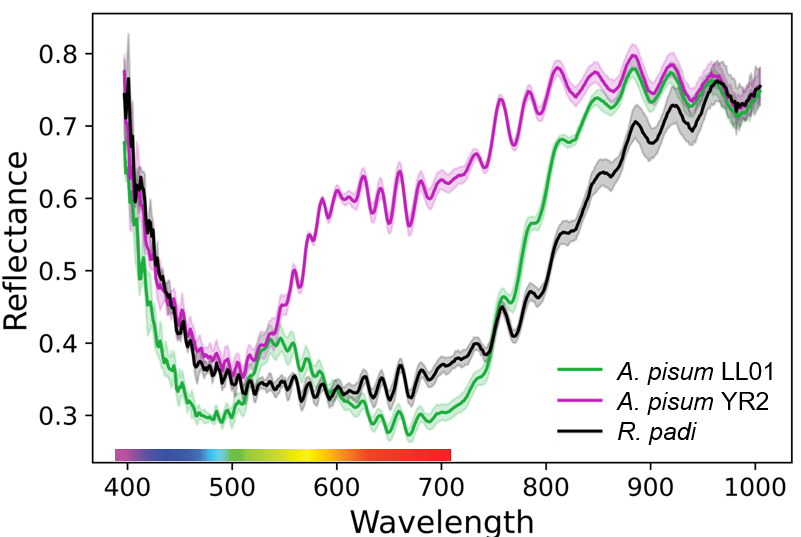


**Supplementary Figure 1**: Average light reflectance spectrums between 400 and 1 000 nm of the three aphid lines. Measurements made with hyperspectral camera Specim® FX10. Aphids absorb the visible part of the light spectrum between 400 and 700 nm but reflect some specific ranges differently, which could explain their different colors (green light area for A. pisum LL01 and red-light area for YR2). However, each of the three aphid lines strongly reflect infrared.

**Supplementary Figure 2**: Average intrinsic rate of increase (r_m_) of the first (F0) and second (F1) aphid generations. F0 C refers to the untreated first generation, while F0 L refers to the irradiated first generation. F1 JX refers to the second generation born the 1^st^, 5^th^ *,10*^th^ *and 15*^th^ *day during the F0 reproduction period. There were five replicates per treatment (sample size too small for ANOVA statistics).*

**
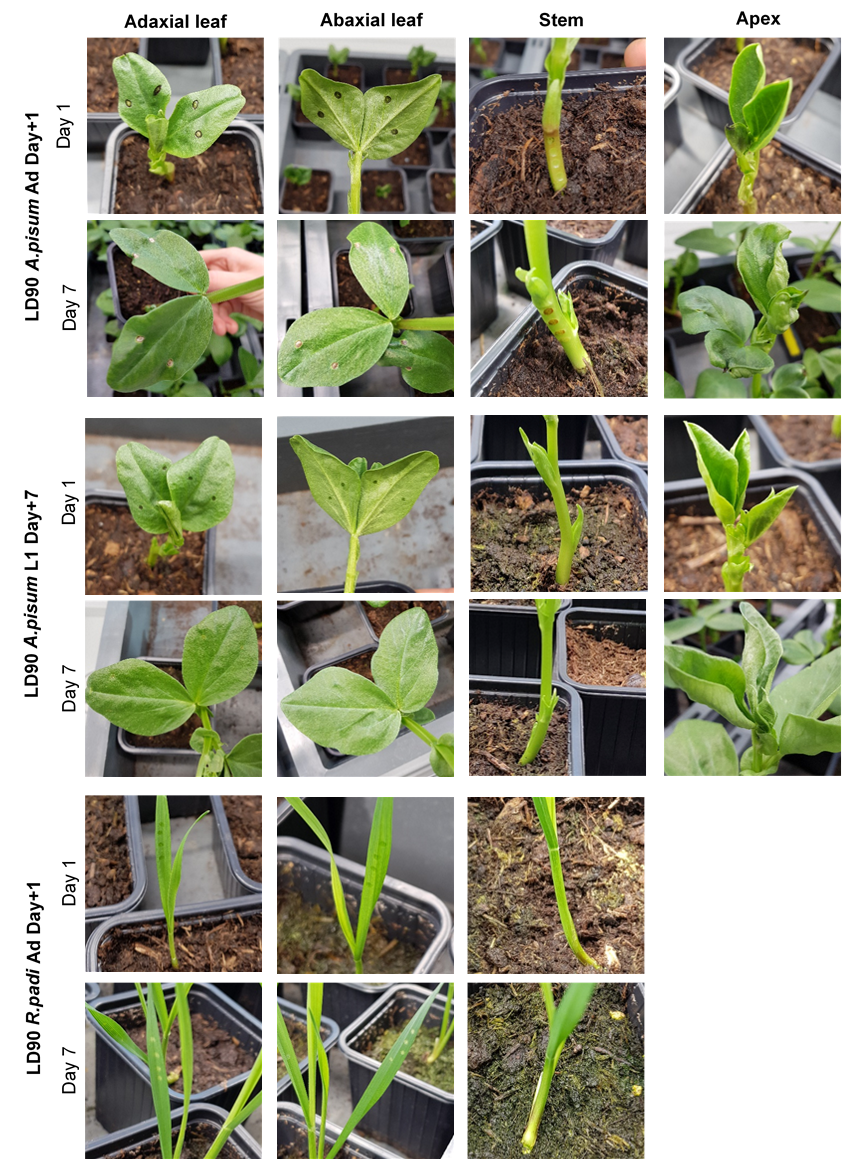
**

**Supplementary Figure 3**: Pictures of irradiated V. faba and T. aestivum plants a few hours and seven days after irradiation. Pictures taken by Virginie Lacotte (BF2i laboratory)

**
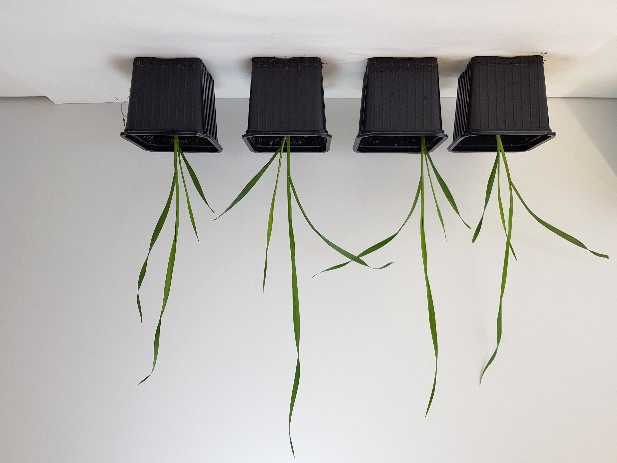

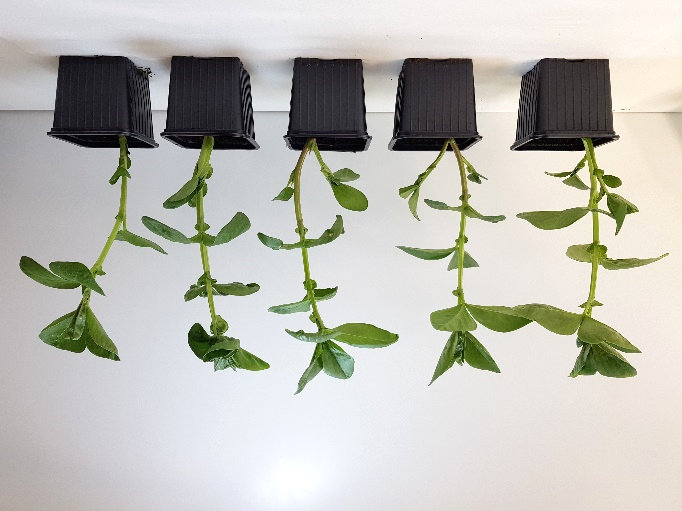
Supplementary Figure 4**: Pictures of V. faba and T. aestivum plants seven days after irradiation at the dose LD90 Day+1 for A. pisum and R. padi adults. a) From left to right: Control; Adaxial leaf surface; Abaxial leaf surface; Apex; Stem. B) From left to right: Control; Adaxial leaf surface; Abaxial leaf surface; Stem. Pictures taken by Virginie Lacotte (BF2i laboratory)


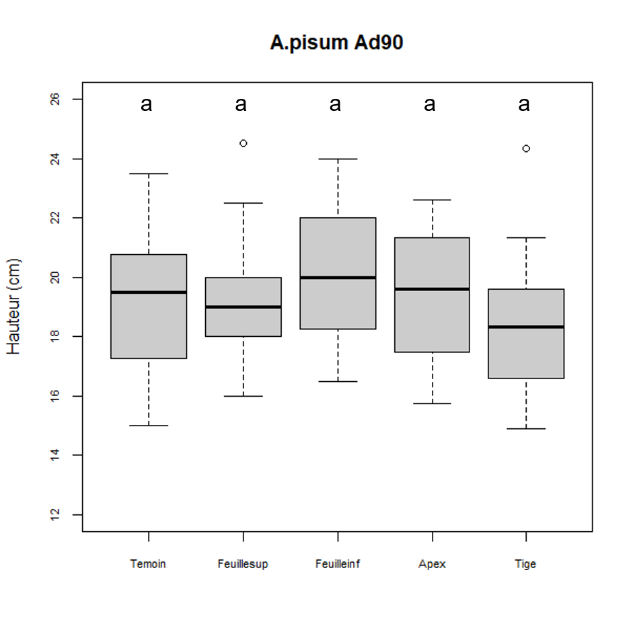

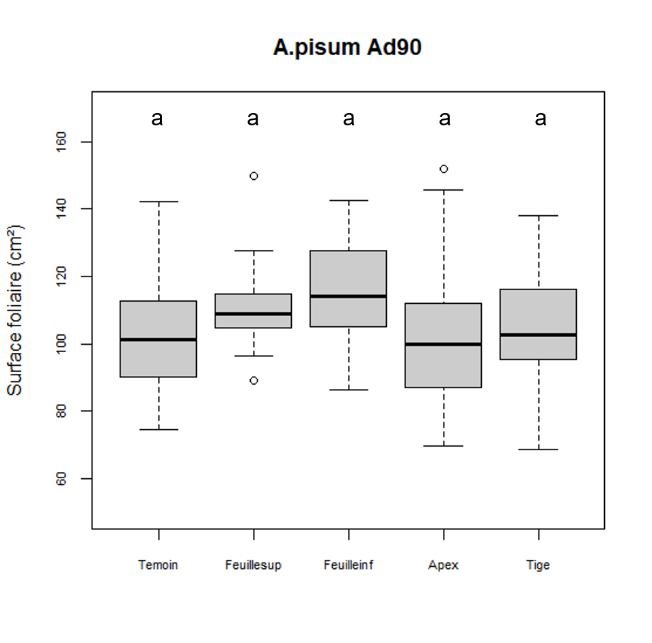

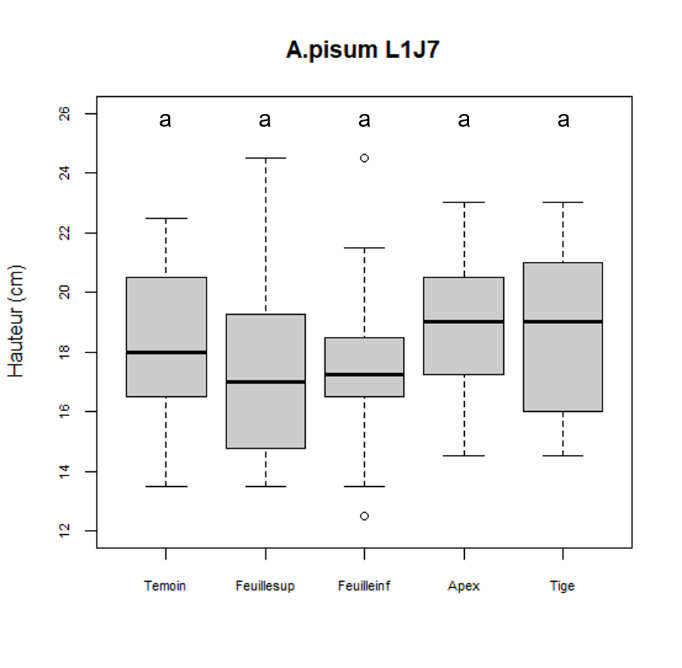

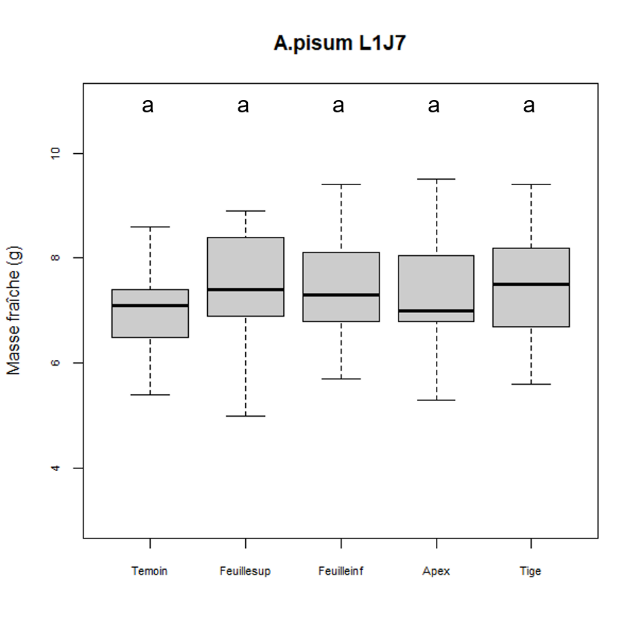

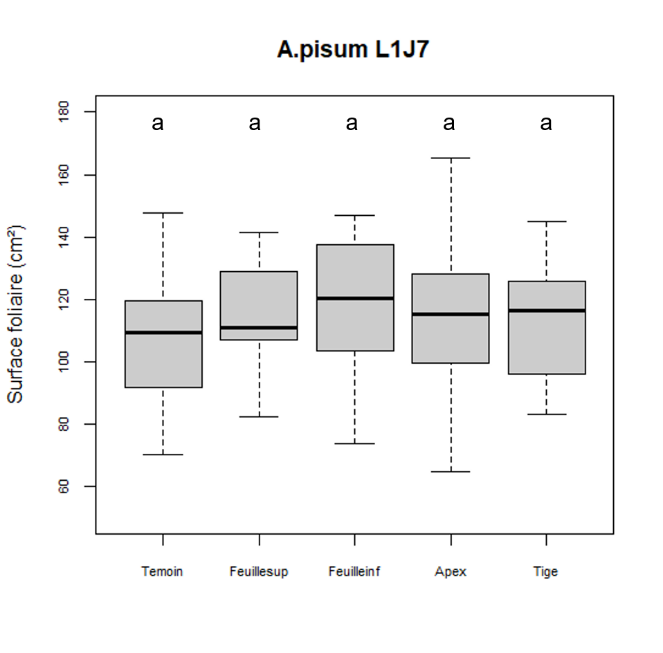

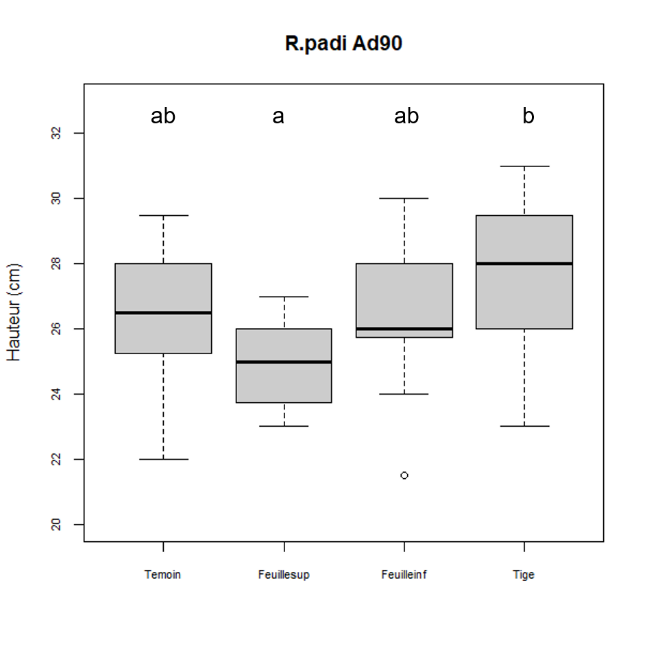

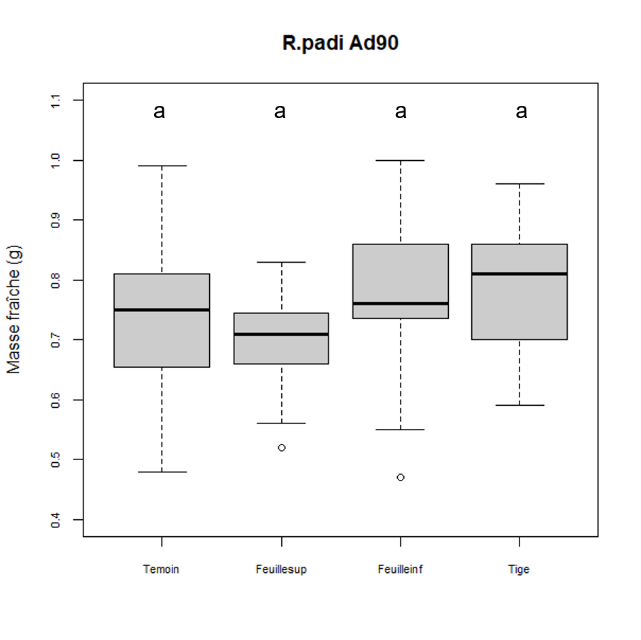

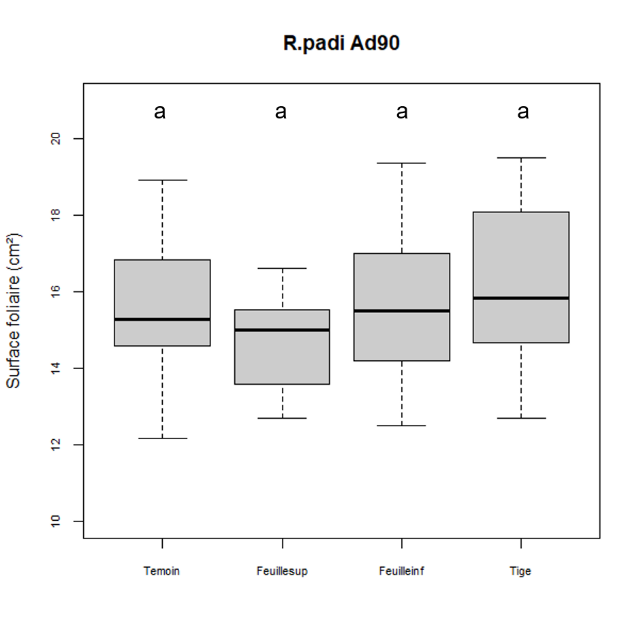

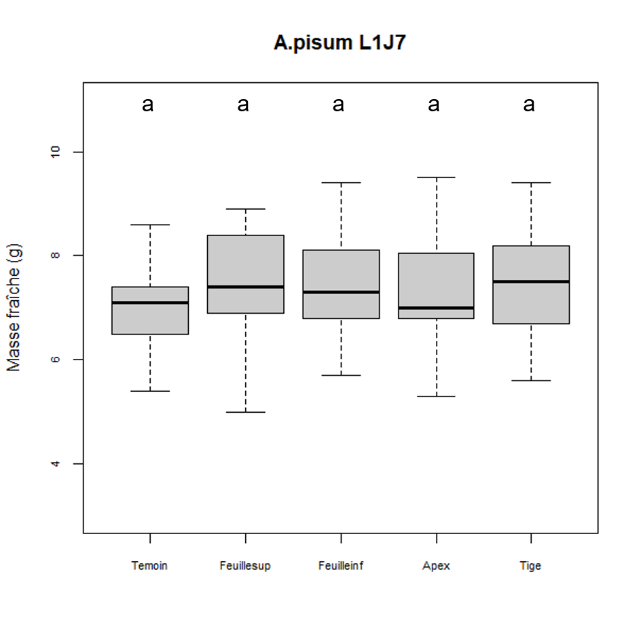

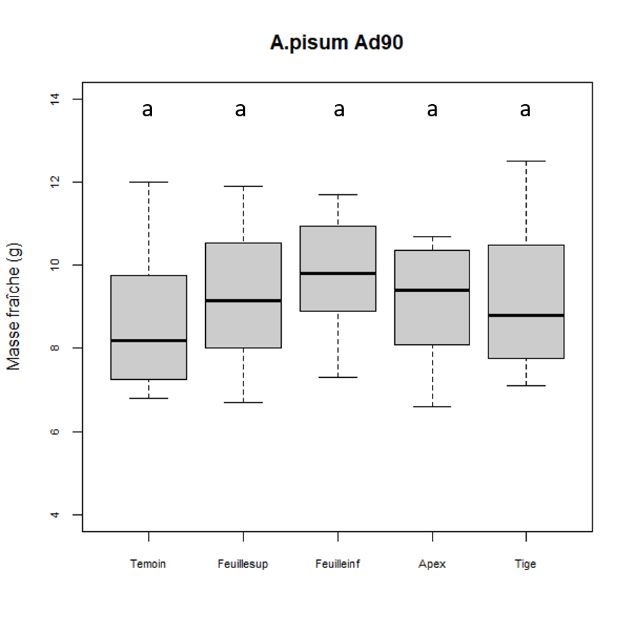


Height (cm)

Fresh mass (g)

Leaf area (cm²)

Control Leaf Leaf Apex Stem

Adaxial Abaxial

Control Leaf Leaf Apex Stem

Adaxial Abaxial

Control Leaf Leaf Stem

Adaxial Abaxial

***A.pisum* AdJ1**

***A.pisum* L1J7**

***R.padi* AdJ1**

**Supplementary Figure 5:** Height (cm), fresh mass (g), foliar area (cm^2^) after seven days of development for irradiated *V. faba* and *T. aestivum* plants. 95% confidence interval ANOVA test.

b)adiated V.faba and T. aestivum plants

a) irradiated V.faba and T. aestivum plants


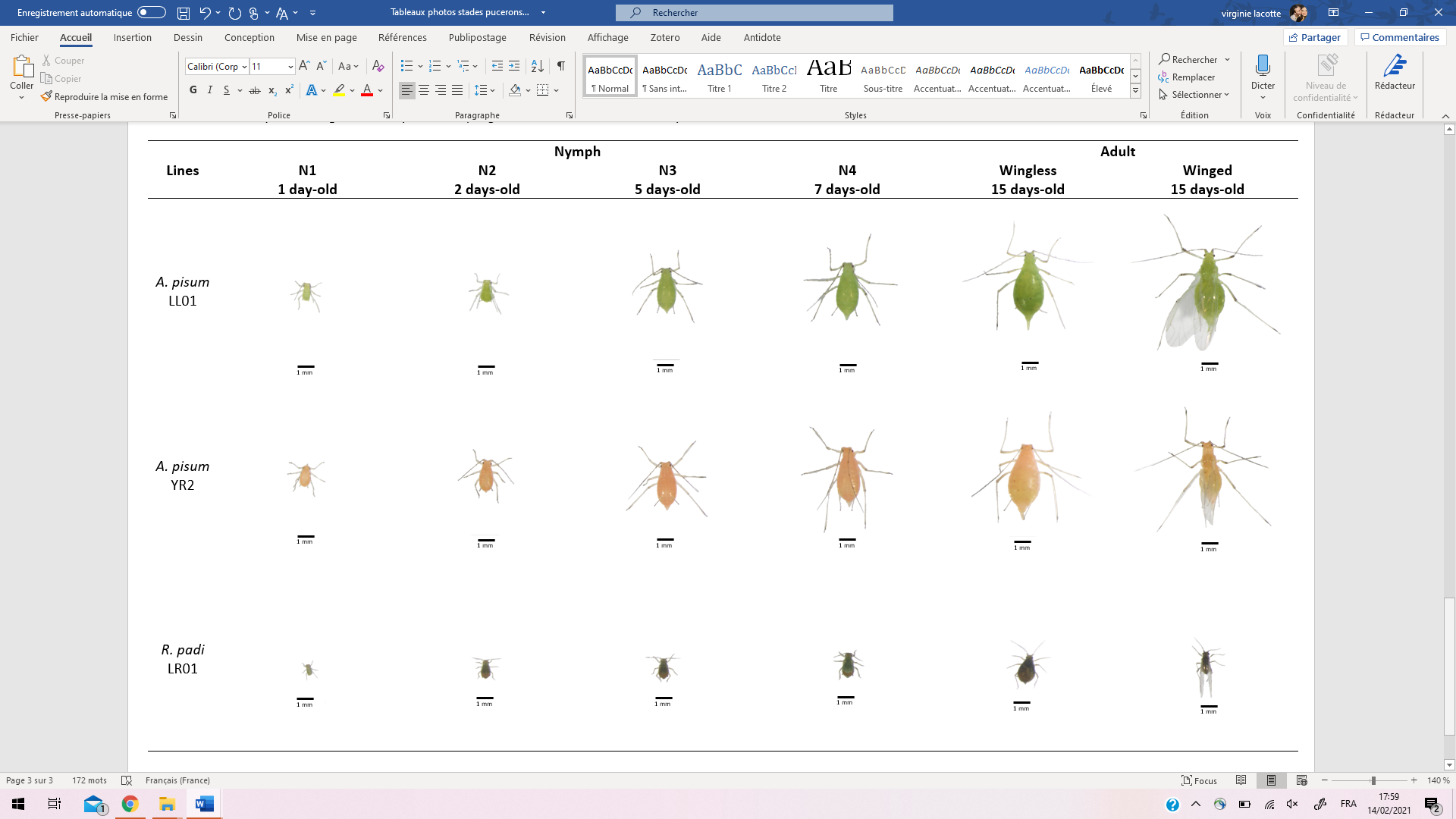
**Supplementary Figure 6:** Development stages of the three aphid lines used in this study. Pictures taken by Virginie Lacotte (BF2i laboratory)


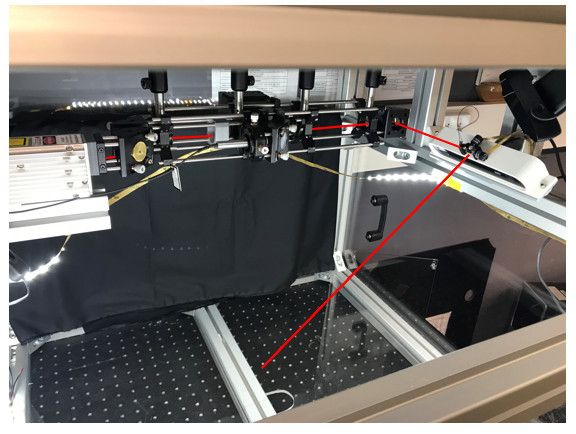


**Supplementary Figure 7:** Picture of the experimental setup. Red line: Beam trajectory. The aphid sample would be placed at the end of the beam, on the dark table.


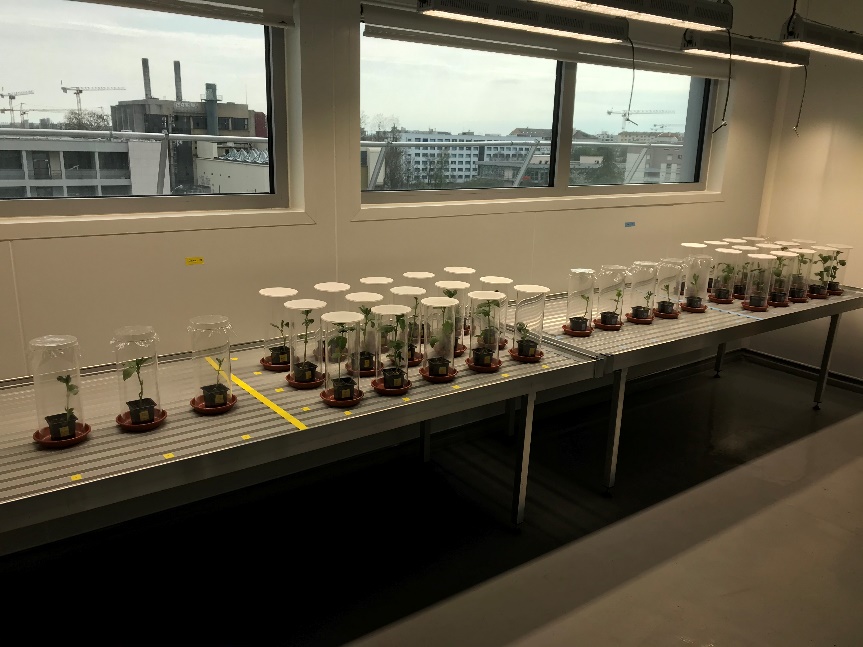

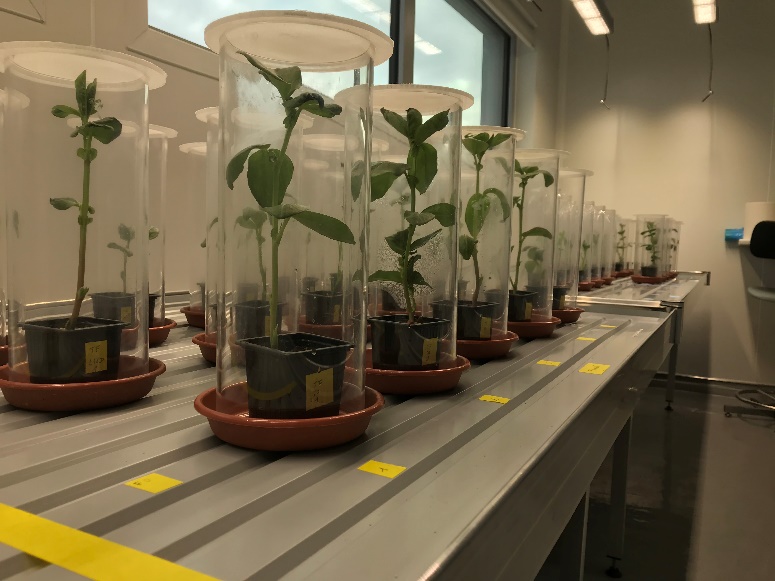


**Supplementary Figure 8:** Pictures of the setup used for the biological monitoring of aphids**.** Pictures taken by Virginie Lacotte (BF2i laboratory)

**Supplementary Figure 9:**
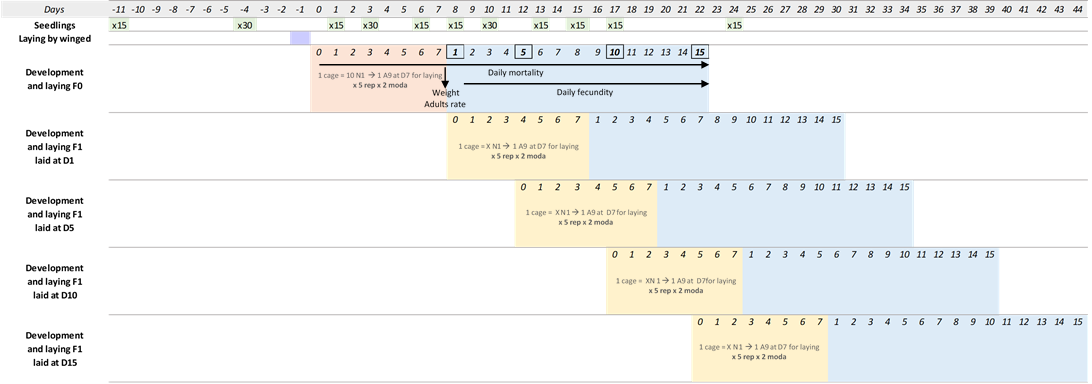
Planning and measures of the biological monitoring of aphids on the generations F0 and F1.
